# Case Report: Angiography-guided transvenous single-chamber pacemaker implantation in mirror-image dextrocardia with congenitally corrected transposition of the great arteries

**DOI:** 10.3389/fcvm.2026.1956397

**Published:** 2026-10-05

**Authors:** Junda Cao, Qin Ao, Jin Ma, Ji Sun

**Affiliations:** 1 Jiujiang City Key Laboratory of Cell Therapy, Department of Cardiovascular Medicine, The First Hospital of Jiujiang City, Jiujiang, China; 2 Department of Cardiovascular Medicine, The First Affiliated Hospital, Sun Yat-sen University, Guangzhou, China

**Keywords:** Mirror-image dextrocardia, Congenitally corrected transposition of the great arteries, Slow ventricular response, Permanent pacemaker, Axillary venography

## Abstract

**Background:**

Mirror-image dextrocardia with situs inversus is uncommon. Transvenous permanent pacemaker implantation is technically demanding when it coexists with congenitally corrected transposition of the great arteries (ccTGA), because venous access, atrioventricular valve crossing, and fluoroscopic lead orientation may differ from usual anatomy.

**Case presentation:**

A 61-year-old man was admitted with recurrent dizziness and palpitations for 5 years; his symptoms had worsened during the preceding 2 weeks. Preoperative 12-lead electrocardiography showed atrial fibrillation with a slow ventricular response. Twenty-four-hour Holter monitoring documented 707 pauses longer than 2.0 s, with a longest R-R interval of 6.13 s. Echocardiography confirmed mirror-image dextrocardia, situs inversus, and ccTGA. A single-chamber permanent pacemaker was implanted through the right axillary vein. Posteroanterior fluoroscopy defined the right-sided cardiac silhouette; right axillary venography guided venous puncture; right atrial angiography identified the morphologic mitral annular plane; and multi-projection fluoroscopy was used to confirm the final ventricular lead position in the high mid-septal region of the morphologic left ventricle. The procedure lasted 56 min and had no acute complications. At 6-month outpatient follow-up, standard 12-lead electrocardiography, 24-hour Holter monitoring, formal device interrogation, and transthoracic echocardiography showed stable pacing, preserved ventricular performance, and no device-related complication; the paced QRS duration was 144 ms and the pacing burden was 99.8%. During telephone follow-up in June 2026, approximately 1 year after implantation, the patient reported no discomfort.

**Conclusion:**

This case describes a structured approach to conventional transvenous pacemaker implantation in mirror-image dextrocardia with ccTGA. In this patient, venography, chamber angiography, and targeted fluoroscopic views reduced procedural uncertainty, and follow-up through 1 year showed clinical stability without recognized complications.

## Introduction

Mirror-image dextrocardia is a rare congenital condition in which the heart lies in the right hemithorax and the thoracoabdominal organs are arranged in a mirror-image pattern. Dextrocardia may be isolated, but it can also occur with clinically important congenital cardiac malformations (1). When permanent pacing is required, the implanting physician must account for an altered cardiac silhouette, venous route, valve orientation, and fluoroscopic lead course (1, 9).

The implantation problem is greater when mirror-image dextrocardia coexists with congenitally corrected transposition of the great arteries (ccTGA). In ccTGA, atrioventricular and ventriculoarterial discordance create a physiologically corrected but anatomically unusual circulation. Previous reports have described pacemaker implantation in patients with dextrocardia using conventional transvenous systems, cardiac resynchronization therapy, or leadless devices (2–7). Fewer reports describe the procedural sequence used to confirm venous access, localize the atrioventricular annulus, and verify the final lead position.

We report a patient with mirror-image dextrocardia, situs inversus, ccTGA, and symptomatic atrial fibrillation with a slow ventricular response who underwent right axillary transvenous single-chamber pacemaker implantation. The report includes 6-month outpatient reassessment and 1-year telephone follow-up.

## Case description

A 61-year-old man was admitted to the Department of Cardiovascular Medicine because of recurrent dizziness and palpitations for 5 years. His symptoms had worsened during the previous 2 weeks. His medical history included chronic obstructive pulmonary disease and benign prostatic hyperplasia. Mirror-image dextrocardia with situs inversus had been identified during a physical examination in 2021. He denied a history of hypertension, diabetes mellitus, or coronary artery disease.

On admission, his temperature was 36.1 degrees C, pulse rate was 50 beats/min, respiratory rate was 19 breaths/min, and blood pressure was 156/79 mmHg. He was alert. Lung auscultation was clear. Cardiac examination revealed an irregular rhythm at approximately 52 beats/min. The abdomen was soft, and no lower-extremity edema was present.

Routine blood gas analysis, complete blood count, liver and renal function tests, electrolytes, myocardial enzymes, lipid profile, coagulation testing, D-dimer, thyroid function, N-terminal pro-B-type natriuretic peptide, troponin T, C-reactive protein, human immunodeficiency virus antibody, syphilis antibody, and cranial magnetic resonance imaging showed no major abnormality that explained the dizziness.

The preoperative 12-lead electrocardiogram, recorded with standard electrode positions, showed atrial fibrillation with a slow ventricular response and ST-T changes (Figure 1A). Twenty-four-hour Holter monitoring demonstrated atrial fibrillation with a slow ventricular response. The mean heart rate was 45 beats/min, the minimum heart rate was 30 beats/min, 707 pauses were longer than 2.0 s, and the longest R-R interval was 6.13 s (Figure 1B). Ventricular premature beats, paired ventricular premature beats, short runs of nonsustained ventricular tachycardia, and ST-T changes were also reported.

**Figure 1:**
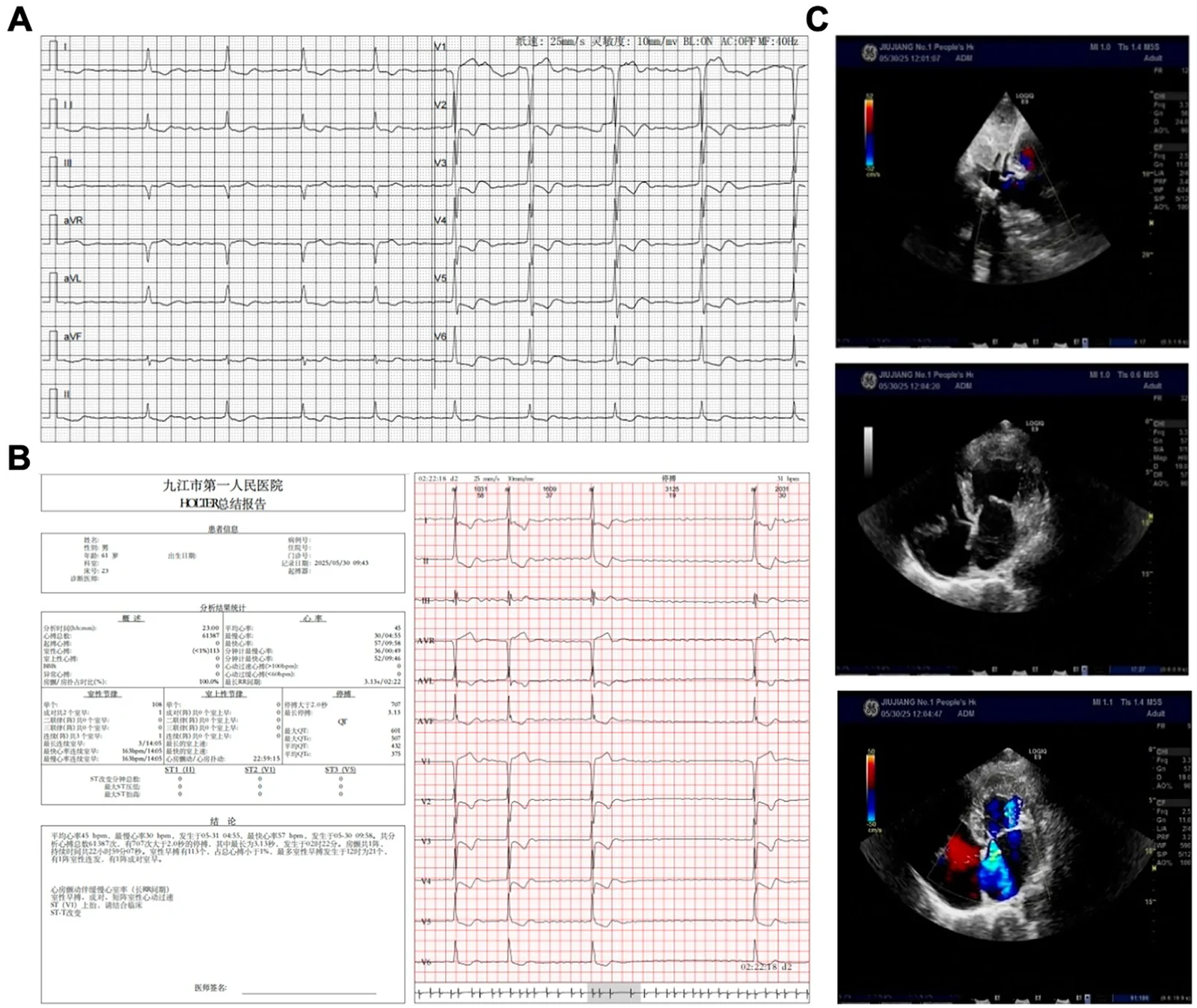
Preoperative evaluation. All 12-lead electrocardiograms were recorded with standard electrode positions. **(A)** Preoperative routine 12-lead electrocardiogram showing atrial fibrillation with a slow ventricular response and ST-T changes. **(B)** Preoperative 24-hour Holter monitoring showing atrial fibrillation with a slow ventricular response, repeated long pauses, and a longest R-R interval of 6.13 s. **(C)** Preoperative transthoracic echocardiography showing mirror-image dextrocardia, situs inversus, and ccTGA.

Transthoracic echocardiography confirmed mirror-image dextrocardia and situs inversus (Figure 1C). The spleen was located in the right upper abdomen, the liver in the left upper abdomen, the abdominal aorta on the right side of the spine, and the inferior vena cava on the left side of the spine. The heart was in the right hemithorax, with the apex pointing rightward and inferiorly. The atria were inverted. Ventricular topology was consistent with ccTGA: the left atrium connected to the morphological right ventricle through the tricuspid valve, and the morphological right ventricle connected to the aorta; the right atrium connected to the morphologic left ventricle through the morphologic mitral valve, and the morphologic left ventricle connected to the pulmonary artery. Ventricular systolic function was preserved, with an ejection fraction of 60.88%. The left atrium measured 42 mm, the ventricular end-diastolic diameter was 48.2 mm, and the pulmonary artery trunk was enlarged to 39 mm. Color Doppler imaging showed moderate mitral and tricuspid regurgitation, mild-to-moderate aortic regurgitation, and mild pulmonary regurgitation. A small atrial septal defect of approximately 5 mm was suspected, but it was not confirmed by contrast or transesophageal echocardiography; no definite right-to-left or bidirectional shunt was documented, and anticoagulation was not prescribed. A small posterior pericardial effusion of approximately 4 mm was noted.

Given the recurrent dizziness, persistent atrial fibrillation with a slow ventricular response, and repeated long pauses, permanent pacing was indicated (10). The clinical timeline is summarized in Table 1.

**Table 1:** Clinical timeline.

Date or period	Clinical event
2021	Mirror-image dextrocardia and situs inversus identified during physical examination.
Previous 5 years	Recurrent dizziness and palpitations.
2 weeks before admission	Symptoms worsened.
May 29, 2025	Preoperative 12-lead electrocardiography showed atrial fibrillation with a slow ventricular response.
May 30, 2025	Holter monitoring showed a mean heart rate of 45 beats/min, 707 pauses longer than 2.0 s, and a longest R-R interval of 6.13 s. Echocardiography confirmed mirror-image dextrocardia, situs inversus, and ccTGA.
June 4, 2025	Single-chamber pacemaker implantation was performed with right axillary venography and right atrial angiography guidance.
Postoperative day 1	Electrocardiography showed ventricular-paced rhythm; device interrogation confirmed stable pacing function.
Approximately 6 months after implantation	Outpatient 12-lead electrocardiography showed stable ventricular-paced rhythm with a paced QRS duration of 144 ms. Device interrogation confirmed VVIR mode, a lower rate of 60 beats/min, a pacing burden of 99.8%, bipolar impedance of 510 ohms, pacing threshold of 0.75 V, and R-wave amplitude of 6.0 mV; transthoracic echocardiography showed no recognized complication.
June 2026	Telephone follow-up approximately 1 year after implantation showed no subjective discomfort.

## Therapeutic intervention

After discussion with the patient and his family, written informed consent for the procedure was obtained. On June 4, 2025, single-chamber permanent pacemaker implantation was performed in a digital subtraction angiography suite. The patient was placed supine, electrocardiographic monitoring was established, and peripheral venous access was secured.

Preoperative posteroanterior fluoroscopy confirmed the right-sided cardiac silhouette and mirror-image orientation (Figure 2A). Right axillary venography showed a suitable venous course and guided puncture planning (Figure 2B). After standard skin preparation and draping, local anesthesia was achieved with 1% lidocaine. The distal right axillary vein was punctured successfully on the first attempt under fluoroscopic guidance. A hydrophilic guidewire was exchanged through a 6F vascular sheath. Right atrial angiography identified the morphologic mitral annular plane and provided an anatomic target for valve crossing into the morphologic left ventricle (Figure 2C).

**Figure 2:**
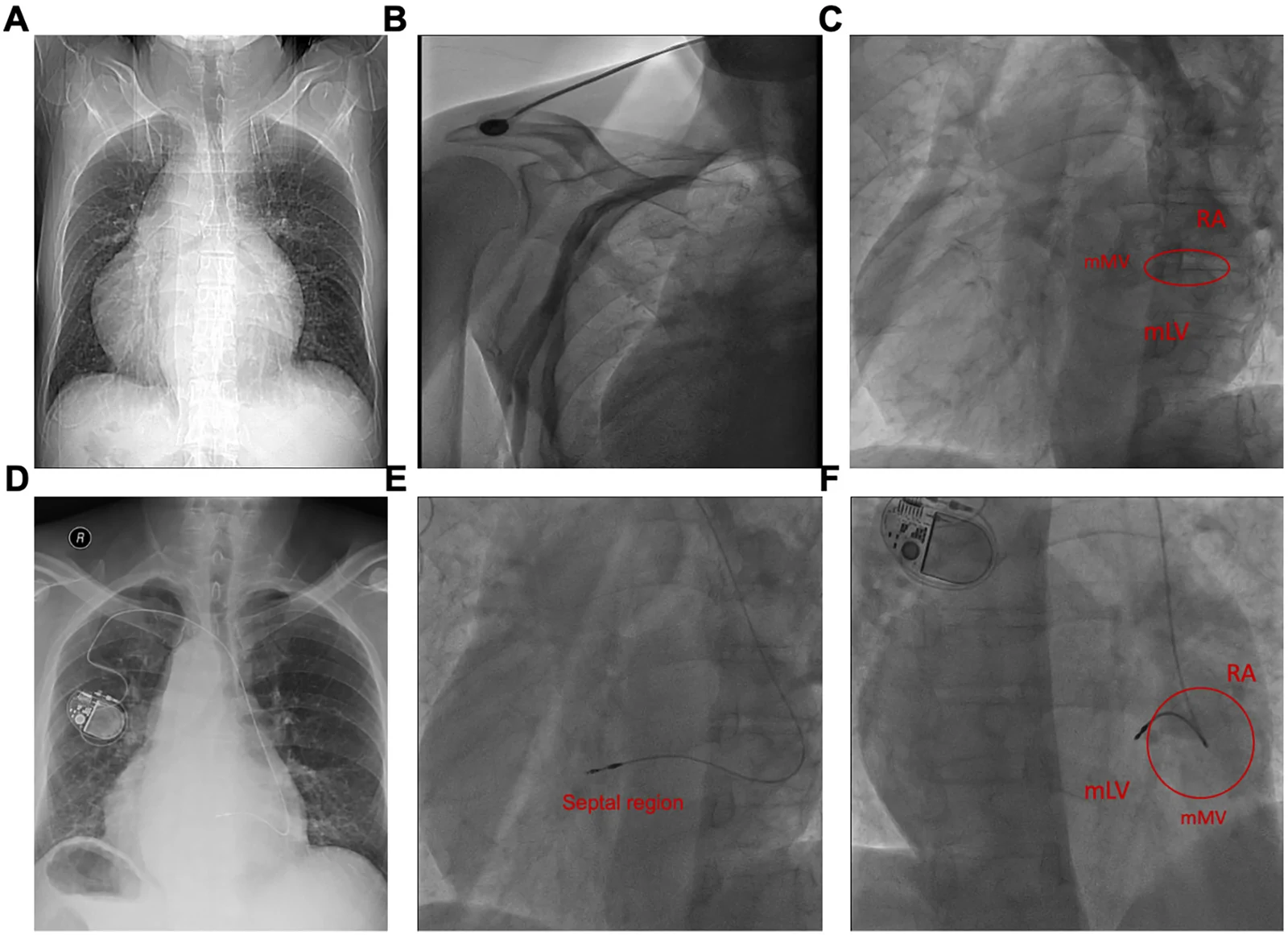
Procedural images. **(A)** Preoperative posteroanterior fluoroscopy confirming the right-sided cardiac silhouette. **(B)** Right axillary venography used to guide venous puncture. **(C)** Right atrial angiography confirming the morphologic mitral annular plane. **(D)** Posteroanterior fluoroscopy after placement of the lead and pacemaker generator. **(E)** Left anterior oblique 30-degree fluoroscopy. **(F)** Right anterior oblique 25-degree fluoroscopy.

A transverse incision of approximately 6 cm was made from the puncture site, and a subcutaneous pocket was created superficial to the pectoral fascia. An 8F peel-away sheath was advanced over the guidewire. A bipolar active-fixation ventricular lead (St. Jude Medical 2088TC-58) was delivered through the sheath and positioned at the high mid-septal region of the morphologic left ventricle. Posteroanterior fluoroscopy after lead and generator placement confirmed the overall system configuration (Figure 2D). Left anterior oblique 30-degree fluoroscopy (Figure 2E) and right anterior oblique 25-degree fluoroscopy (Figure 2F) further verified the lead course and final position.

The measured bipolar lead impedance was 510 ohms, the pacing threshold was 0.8 V, and the acute R-wave amplitude was 7.0 mV. The lead was fixed to the subcutaneous tissue with sutures and connected to a single-chamber pacemaker generator (St. Jude Medical 1282 MRI). The generator and lead were placed in the pocket, and the incision was closed layer by layer. The pacemaker was programmed in VVIR mode with a lower rate of 60 beats/min. The procedure lasted 56 min and was completed without acute discomfort or procedural instability. A sandbag compression dressing was applied for 6 h, followed by routine postoperative pacemaker care.

## Follow-up and outcomes

Postoperative 12-lead electrocardiography obtained with standard electrode positions, shown in Figure 3A, demonstrated a ventricular-paced rhythm and provided the postoperative reference rhythm for comparison with follow-up recordings. Device interrogation on postoperative day 1 showed satisfactory pacemaker function, and no pauses longer than 2.0 s were observed. Postoperative day 2 transthoracic echocardiography showed the lead tip fixed in the high mid-septal region of the morphologic left ventricle. The patient's dizziness improved. No pocket hematoma, pneumothorax, lead dislodgement, device malfunction, or other pacemaker-related complication occurred during the in-hospital observation period.

**Figure 3:**
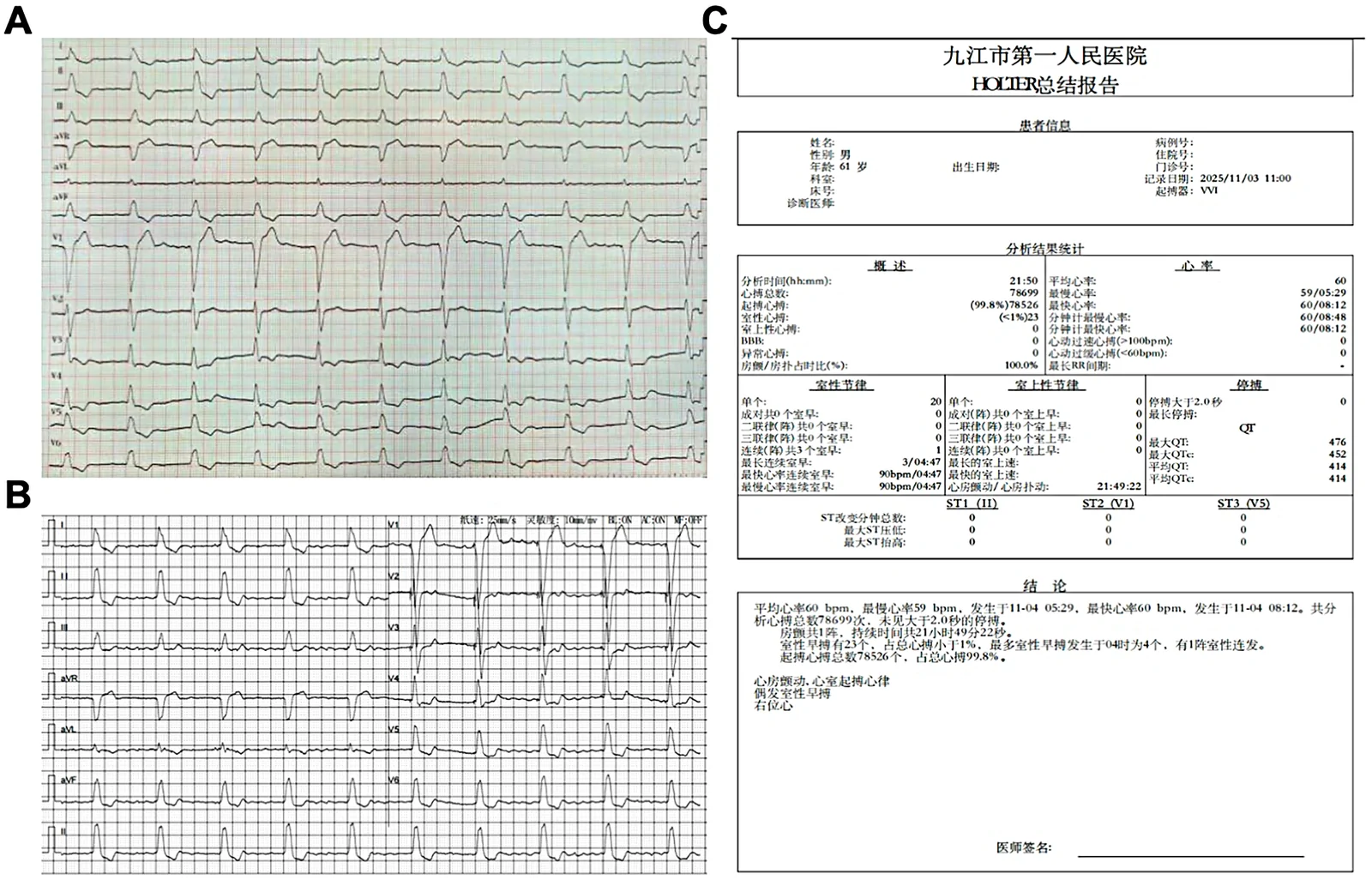
Postoperative and follow-up evaluation. All 12-lead electrocardiograms were recorded with standard electrode positions. **(A)** Postoperative routine 12-lead electrocardiogram showing ventricular-paced rhythm. **(B)** Routine 12-lead electrocardiogram at 6-month follow-up showing stable ventricular-paced rhythm with a paced QRS duration of 144 ms. **(C)** Twenty-four-hour Holter monitoring at 6-month follow-up showing a paced rhythm at approximately 60 beats/min and no pauses longer than 2.0 s.

The patient returned for outpatient follow-up approximately 6 months after implantation. Routine 12-lead electrocardiography, obtained with standard electrode positions, showed a stable ventricular-paced rhythm with a paced QRS duration of 144 ms (Figure 3B). Twenty-four-hour Holter monitoring showed a paced rhythm at approximately 60 beats/min and no pauses longer than 2.0 s (Figure 3C). Formal device interrogation at the same visit confirmed VVIR mode, a lower rate of 60 beats/min, bipolar impedance of 510 ohms, pacing threshold of 0.75 V, R-wave amplitude of 6.0 mV, and a pacing burden of 99.8%. Transthoracic echocardiography showed preserved ventricular systolic function (ejection fraction 58%), left ventricular end-diastolic diameter 47 mm, left atrial diameter 42 mm, pulmonary artery trunk diameter 38 mm, and persistent moderate mitral and tricuspid regurgitation with mild-to-moderate aortic regurgitation and mild pulmonary regurgitation, without new device-related complication. The patient reported no recurrent dizziness, syncope, pocket discomfort, or other procedure-related symptom. During telephone follow-up in June 2026, approximately 1 year after implantation, he reported no subjective discomfort.

## Discussion

This case describes transvenous pacing in a patient with mirror-image dextrocardia, situs inversus, ccTGA, and symptomatic atrial fibrillation with long pauses. Three aspects are clinically relevant: intraprocedural angiography to reduce anatomic uncertainty, multi-view fluoroscopic verification of the lead tip in thehigh mid-septal region of the morphologic left ventricle, and follow-up that extended from 6-month in-person assessment to 1-year telephone contact.

Mirror-image dextrocardia may resemble a simple left-right reversal, but ccTGA changes the procedural anatomy. The operator must account for atrial inversion, discordant atrioventricular connections, and an unfamiliar relationship between the lead tip and the ventricular septum. In this setting, a routine posteroanterior view alone may be insufficient. In our case, preoperative fluoroscopy established the global right-sided orientation (Figure 2A). Venography and chamber angiography then mapped the access route and annular target (Figures 2B,C).

Right axillary venography confirmed a suitable puncture route before pocket creation and sheath advancement. This step may be useful in dextrocardia or situs inversus, where associated venous anomalies can complicate an otherwise routine device procedure. Right atrial angiography localized the morphologic mitral annular plane and guided lead manipulation across the atrioventricular valve into the morphologic left ventricle. Together, these angiographic steps provided a procedural map specific to the patient's anatomy.

After placement of the ventricular lead and generator, posteroanterior, left anterior oblique 30-degree, and right anterior oblique 25-degree fluoroscopy confirmed the device configuration and lead course (Figures 2D–F). The final lead position was supported by the fluoroscopic trajectory, intracardiac response during manipulation, electrical parameters, and postoperative day 2 transthoracic echocardiography showing the lead tip in the high mid-septal region of the morphologic left ventricle. The acute impedance, capture threshold, and R-wave amplitude were satisfactory, consistent with secure initial fixation in thehigh mid-septal region of the morphologic left ventricle.

Follow-up is important in patients with ccTGA because systemic ventricular dysfunction, atrioventricular valve regurgitation, and conduction disease can evolve over time. The 6-month outpatient assessment in this patient included routine 12-lead electrocardiography, 24-hour Holter monitoring, device interrogation, and transthoracic echocardiography. These examinations showed stable pacing, preserved ventricular systolic function, and no recognized device-related complication. At the 1-year telephone follow-up, the patient reported no discomfort.

Earlier case reports established that permanent pacing is feasible in mirror-image dextrocardia (2). Later reports and small series included transvenous pacing, leadless pacing, and resynchronization therapy in different dextrocardia variants (3–8). A concise comparison of selected prior reports is provided in Table 2. The present case does not claim first-in-human novelty. Its contribution is a stepwise procedural description: confirming the global mirror-image orientation, defining venous access with axillary venography, identifying the annular target with right atrial angiography, and verifying the final lead course in multiple fluoroscopic projections. Retrospectively, leadless pacing and conduction-system pacing were considered as alternatives; however, because of the markedly altered anatomy, we attempted to identify the His-bundle region intraoperatively but could not localize a suitable target, and conventional transvenous active-fixation pacing was therefore selected. The leadless option was financially out of reach for the patient and family, while the transvenous route was feasible and provided reliable electrical parameters. At 6 months, the paced QRS duration of 144 ms and pacing burden of 99.8% supported effective pacing but also reinforced the need for continued surveillance in ccTGA. The 6-month in-person and 1-year telephone follow-up provided clinical context to the technical report.

**Table 2:** Comparison with selected prior reports.

Study	Anatomic setting	Access route	Device type	Ventricular target	Imaging guidance	Complications/follow-up
Goyal et al., 1976 2	Mirror-image dextrocardia; double SVC; absent IVC with azygos continuation	Left superior vena cava via cephalic vein	Bipolar demand pacemaker	Right ventricular apex	Angiographic studies; lateral angiographic projection	Uneventful recovery; symptom-free discharge at 1 week
Shenthar et al., 2014 3	Six cases: 3 situs inversus, 3 situs solitus with ccTGA	Right-sided implantation; cephalic vein or extra-thoracic axillary puncture; right femoral vein temporary pacing	Permanent pacemaker with active-fixation leads	RV apex in situs inversus; morphologic LV in situs solitus/ccTGA	AP fluoroscopy in all; LAO 30° and RAO 40°; angiography in difficult CC-TGA cases	Mean follow-up 3.9 years (4 months to 7 years); 1 generator replacement
Bontempi et al., 2023 4	Dextrocardia with situs viscerum inversus; TBS; permanent AF; biatrial enlargement	Double right femoral venous access	Micra VR leadless pacemaker	Apical RV septum	Multiplanar fluoroscopy; IVC phlebography; right ventriculography	Uneventful discharge; stable pre-discharge parameters
Bao et al., 2026 5	Mirror-image dextrocardia with hepatic IVC interruption and azygos continuation	Left internal jugular vein	Micra AV leadless pacemaker	Mid-lower ventricular septum	CTA; image mirroring; sheath angiography	No displacement; stable parameters; symptom-free at 1 month
Malecka et al., 2010 6	Dextrocardia with ccTGA and systemic right ventricle	Transvenous CRT approach (access route not specified in abstract)	Three-lead CRT system	Systemic right ventricle/CRT lead placement	Multi-slice CT plus transvenous imaging	20-month follow-up with improved NYHA class, systemic RV EF, and exercise capacity
Aizawa et al., 2021 7	Dextrocardia with situs inversus; advanced AV block	Temporary pacing via right internal jugular vein; permanent LP via femoral vein	Micra VR leadless pacemaker	Septum close to the apex of the right ventricle	Chest radiograph; CT; right ventriculography; RAO/LAO fluoroscopy	Uneventful discharge; next-day threshold improved to 0.38 V
Ciftci et al., 2019 8	Situs inversus dextrocardia with ccTGA after prior repair of ASD/VSD/PS	Left subclavian vein	Endocardial VDD pacemaker	Apex of the morphologic left ventricle	AP fluoroscopy; RAO 40° confirmation	No postoperative complications; normal functional capacity
Present case	Mirror-image dextrocardia, situs inversus, ccTGA, and symptomatic AF with slow ventricular response	Right axillary vein	Single-chamber transvenous pacemaker	High mid-septal region of the morphologic left ventricle	Right axillary venography; right atrial angiography; LAO 30°/RAO 25° fluoroscopy	No acute complication; 6-month device follow-up stable

NR,  not reported in the cited article or available source materials.

This is a single case and cannot define the optimal approach for all patients with mirror-image dextrocardia and ccTGA. Preoperative computed tomography angiography was not available in the provided materials, so the anatomic assessment relied on echocardiography and intraprocedural imaging. The June 2026 follow-up was by telephone only and did not include in-person device interrogation. Longer follow-up with serial device parameters, pacing burden, systemic ventricular function, and atrioventricular valve regurgitation would further clarify long-term safety.

## Patient perspective

At the 6-month outpatient visit, the patient reported no recurrent dizziness, syncope, pocket discomfort, or other procedure-related symptom. During telephone follow-up in June 2026, approximately 1 year after implantation, he reported no subjective discomfort.

## Conclusion

Conventional transvenous single-chamber pacemaker implantation can be performed in selected patients with mirror-image dextrocardia and ccTGA when procedural planning is based on careful anatomic confirmation. In this patient, right axillary venography, right atrial angiography, and multi-projection fluoroscopy supported lead delivery to the high mid-septal region of the morphologic left ventricle and stable pacing parameters. Six-month in-person follow-up showed stable pacing and no recognized complications; the 1-year telephone follow-up provided symptom information only.

## Data Availability

The de-identified clinical data supporting this case report are included in the article. Additional de-identified information may be available from the corresponding author upon reasonable request and with appropriate institutional permission.
